# Comparative study on left-sided versus right-sided hepatectomy for resectable peri-hilar cholangiocarcinoma: a systematic review and meta-analysis

**DOI:** 10.1186/s12957-023-03037-2

**Published:** 2023-05-18

**Authors:** Bowen Xu, Wei Zhao, Jianhua Chang, Jinghua Yin, Nan Wang, Zhaoru Dong, Xuting Zhi, Tao Li, Zhiqiang Chen

**Affiliations:** 1 Department of Hepatobiliary Surgery, National Cancer Center/National Clinical Research Center for Cancer/Cancer Hospital, Chinese Academy of Medical Sciences and Peking Union Medical College, Beijing, 100021 People’s Republic of China; 2 Department of Hepatobiliary Surgery, General Surgery, Qilu Hospital, Cheeloo College of Medicine, Shandong University, 107 West Wen Hua Road, Jinan, 250012 People’s Republic of China; 3 National Engineering Laboratory of Medical Implantable Devices, Key Laboratory for Medical Implantable Devices of Shandong Province, WEGO Holding Company Limited, Weihai, 264210 People’s Republic of China; 4 Department of Hepatobiliary Surgery, General Surgery, The Second Hospital of Shandong University, Jinan, 250033 People’s Republic of China

**Keywords:** Peri-hilar cholangiocarcinoma, Left-sided hepatectomy, Right-sided hepatectomy, Surgical strategy, Survival analysis, Prognosis

## Abstract

**Background:**

Peri-hilar cholangiocarcinoma (pCCA) is a unique entity, and radical surgery provides the only chance for cure and long-term survival. But it is still under debate which surgical strategy (i.e., left-sided hepatectomy, LH or right-sided hepatectomy, RH) should be followed and benefitted.

**Methods:**

We performed a systematic review and meta-analysis to analyze the clinical outcomes and prognostic value of LH versus RH for resectable pCCA. This study followed the PRISMA and AMSTAR guidelines.

**Results:**

A total of 14 cohort studies include 1072 patients in the meta-analysis. The results showed no statistical difference between the two groups in terms of overall survival (OS) and disease-free survival (DFS). But compared to the LH group, the RH group exhibited more employment of preoperative portal vein embolization (PVE), higher rate of overall complications, post-hepatectomy liver failure (PHLF), and perioperative mortality, while LH was associated with higher frequency of arterial resection/reconstruction, longer operative time, and more postoperative bile leakage. There was no statistical difference between the two groups in terms of preoperative biliary drainage, R0 resection rate, portal vein resection, intraoperative bleeding, and intraoperative blood transfusion rate.

**Conclusions:**

According to our meta-analyses, LH and RH have comparable oncological effects on curative resection for pCCA patients. Although LH is not inferior to RH in DFS and OS, it requires more arterial reconstruction which is technically demanding and should be performed by experienced surgeons in high-volume centers. Selectin of surgical strategy between LH and RH should be based on not only tumor location (Bismuth classification) but also vascular involvement and future liver remnant (FLR).

**Supplementary Information:**

The online version contains supplementary material available at 10.1186/s12957-023-03037-2.

## Introduction

Cholangiocarcinoma (CCA) is the second most common malignancy of hepatobiliary system [1] and is categorized into intra-hepatic CCA (iCCA), peri-hilar CCA (pCCA), and distal CCA (dCCA) according to the new ICD-O classification [2]. Nowadays, major hepatectomies with extension to segment I, lymphadenectomy, biliary, and vascular reconstruction all together become the mainstay of surgical strategy for patients with pCCA [3–6]. Bismuth-Corlette classification is broadly used for preoperative assessment of surgical planning and the predominant side of tumor location mostly determines surgical strategy [7]. For example, right-sided hepatectomy (RH, including right heme-hepatectomy and right trisectionectomy) and left-sided hepatectomy (LH, including left hemi-hepatectomy and left trisectionectomy) are mostly accepted as surgical choice for Bismuth type IIIa and IIIb pCCA, respectively. But for Bismuth type I/II/IV tumors, the choice between RH and LH becomes more complicated and controversial, especially when tumors invade to a similar level of both sides of bile duct and both LH and RH could obtain a R0 resection.

Traditionally, more surgeons prefer RH to LH as surgical strategy for pCCA treatment because RH possesses some anatomical advantages over LH for achieving more resection radicality, but it increases the possibility of post-hepatectomy liver failure (PHLF) and mortality. Although LH could preserve more future liver remnant (FLR) volume to decrease PHLF, it bears risks of radicality deficiency and potential complications from vascular reconstruction and multiple anastomoses.

Thus, the purpose of this meta-analysis was to analyze the advantages and disadvantages of LH versus RH in the treatment of pCCA from multiple perspectives using published data, aiming to provide evidence-based strategy on clinical decision making for surgically resectable patients.

## Materials and methods

This study followed the PRISMA and AMSTAR guidelines, which are explicit protocols for systematic reviews and meta-analyses [8–10].

### Search strategies

The Medline, PubMed, Web of Science, Scopus, and Embase databases were searched for eligible studies from inception to December 2021. The following MeSH terms such as “Hepatectomy” and “Klatskin tumour” were used and combined with free terms. Furthermore, the reference lists of relevant literatures were manually cross searched to ensure that all eligible studies were included. All searches were performed independently by two experienced researchers.

### Inclusion and exclusion criteria

The inclusion criteria were regarded as (1) randomized controlled trials (RCT), case–control studies, or cohort studies and (2) perioperative and survival outcomes between RH and LH for the treatment of pCCA should be reported. The exclusion criteria were (1) case reports, reviews, meta-analyses, expert comments, and letters; (2) studies that did not report interested outcomes or survival data could not be extracted; and (3) patients suffered from other biliary duct carcinomas such as iCCA, dCCA, and gallbladder carcinoma.

### Data extraction and literature quality assessment

After eliminating duplicates, two reviewers independently read titles and abstracts. Final studies that met the inclusion and exclusion criteria were identified by reading full text after above steps. Two independent researchers used standardized Excel sheets to extract the following parameters from each study: (1) basic information of the study: first author, year of publication, title, study design, sample size, and duration of follow-up; (2) demographics and perioperative data including age, gender, laboratory tests, Bismuth classification, preoperative biliary drainage (endoscopic biliary drainage, EBD and percutaneous biliary drainage, PBD), future liver remnant (FLR) volume, operating time, blood loos, surgical procedures (RH and LH), transfusion rate, R0 resection rate, portal vein resection (PVR), arterial resection and reconstruction (AR), postoperative morbidity (Clavien-Dindo classification), PHLF, procedure-related mortality, and hospitalization; and (3) prognostic information including overall survival (OS) time, 1-year, 3-year, and 5-year OS rate; disease-free survival (DFS); and 1-year, 3-year, and 5-year DFS rate. The primary outcome of the analysis was OS. Newcastle–Ottawa Scale (NOS) was used to assess risk of bias in non-RCTs.

### Statistical analyses

The meta-analyses were performed using the STATA software (Version 16.0, Stata Corp LP, College Station, TX). Comparisons of OS and DFS were conducted by using hazard ratio (HR) with 95% CI (confidence intervals). If HR were not provided by the original studies, they were extracted from Kaplan–Meier curves by using Engauge Digitizer (version 10.8) or calculated according to the method described by Tierney et al. [11]. Continuous variables were expressed as mean ± standard deviation (SD), and data were transformed if the original study provided only median and interquartile ranges [12, 13]. Dichotomous variables were described by using the odds ratios (OR) with 95% CI.

Heterogeneities between each study were assessed using a chi-square (*χ*^2^) *Q* test. Fixed-effects model was used when heterogeneity was low (*I*^2^ < 50%); otherwise, random-effects model was used (*I*^2^ ≥ 50%). In addition, sources of heterogeneity were attempted to be identified via subgroup analyses and meta-regressions. The publication bias was assessed by Egger’s test and plot funnel. A value of *P* < 0.05 was considered statistically significant.

## Results

**Fig. 1:**
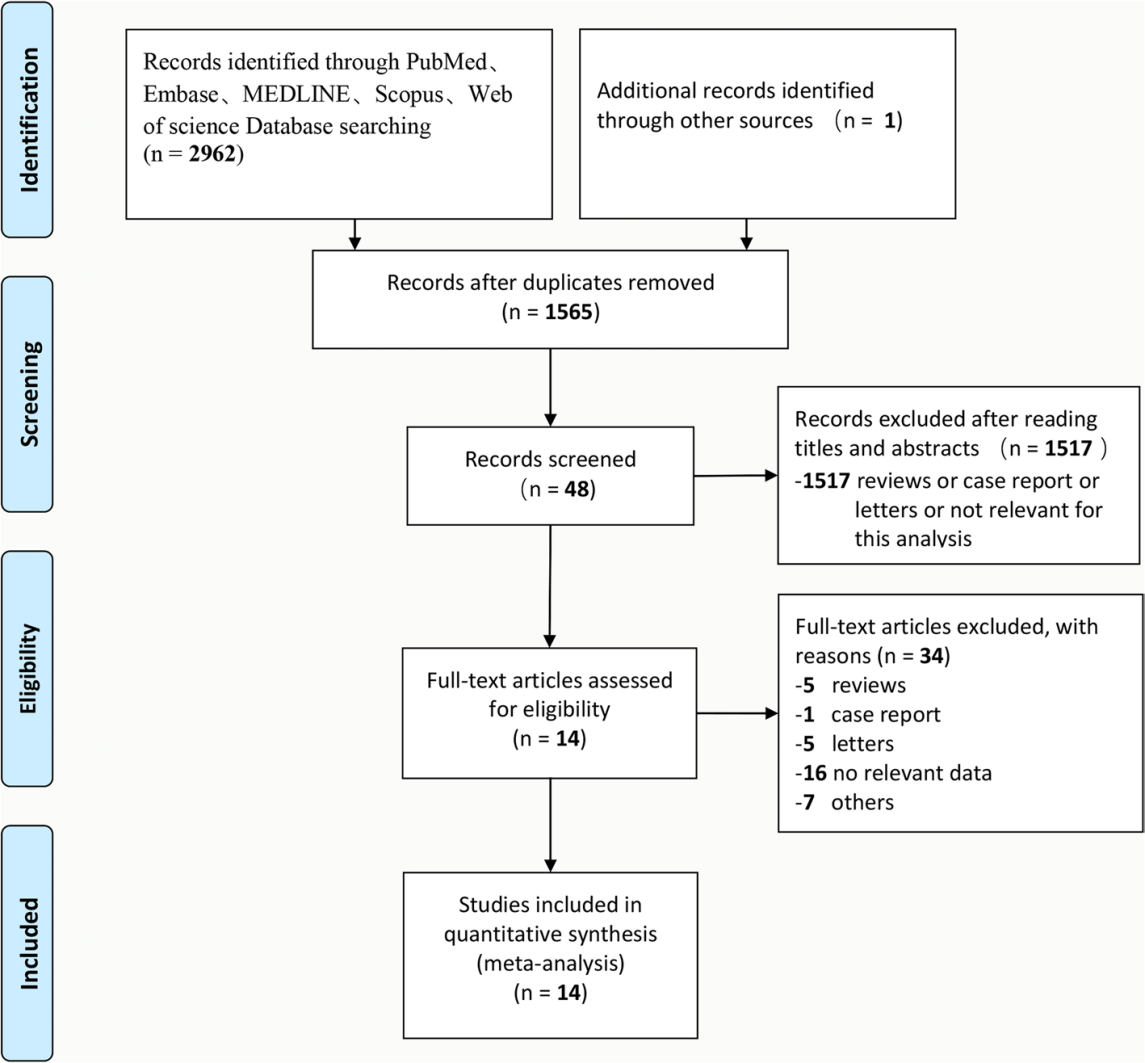
PRISMA flow diagram of the search strategy for studies included in this meta-analysis

A total of 2962 manuscripts were initially identified through 5 databases. After removal of duplicates, titles and abstracts of the remaining 1565 manuscripts were scanned, and 48 studies were included for full-text screening. Eventually, total 14 cohort studies [14–27] were included in the meta-analysis, including 1072 patients of 447 LH and 625 RH (Fig. 1). Four studies [14, 21, 22, 25] originated from western centers (Germany, USA, and Italy) and ten studies [15–20, 23, 24, 26, 27] from eastern centers (Japan, Korea, and India) with all of them being single-center studies. The NOS score of all included studies were 7.0 ± 0.76 with low risk of bias and high quality of evidence. The basic characteristics of all studies were shown in Table 1.

**Table 1:** Basic characteristics of included studies

Reference no, publication year	Country/study period	Patients (*n*)		Bismuth type (*n*) (I/II/III/IV)		Biliary drainage (*n*) (EBD/PBD)		PVE (*n*)		VR (*n*) (PVR/AR)		Median OS (month)		R0 resection *n* (%)		NOS score
		LH	RH	LH	RH	LH	RH	LH	RH	LH	RH	LH	RH	LH	RH	
Bednarsch [14] 2020	Germany/2011–2016	36	45	1/0/23/12	2/6/23/14	27/8	35/11	0	37	36/5	45/0	34	45	25 (69.4)	34 (75.6)	7
Hong [15] 2020	Korea/2000–2018	82	114	5/6/43/28	4/13/75/22	60	93	2	45	4/6	8/1	NR	NR	62 (75.6)	83 (72.8)	8
Jo [16] 2020	Korea/2010–2017	24	33	NR/NR/NR/7	NR/NR/NR/12	14/8	20/9	0	6	2/NR	9/NR	NR	NR	18 (75.0)	25 (75.8)	8
Hosokawa [17] 2019	Japan/2008–2016	27	27	NR/2/8/17	NR/2/9/16	NR	NR	NR	NR	NR	NR	NR	NR	15 (55.6)	12 (44.4)	8
Sugiura [18] 2019	Japan/2002–2013	12	24	2/10/NR/NR	8/16/NR/NR	NR	NR	0	24	1/NR	4/NR	44	57	11 (91.7)	22 (91.7)	7
Lee [19] 2018	Korea/1995–2012	35	103	NR/NR/35/NR	NR/NR/103/NR	23	71	0	24	NR	NR	36		30 (85.7)	85 (82.5)	8
Govil [20] 2016	India/2009–2015	23	13	NR/NR/15/8	NR/NR/13/0	0/8	0/6	0	0	15/10	4/0	22	20	29 (87.9)	12 (92.3)	6
Ratti [21] 2015	Italy/2004–2014	44	61	1/17/13/13	1/20/15/25	6/16	9/22	0	29	7/5	10/7	NR	NR	27 (61.4)	46 (75.4)	7
Otto [22] 2012	Germany/1998–2011	68	68	NR	NR	NR	NR	0	4	NR	NR	26	37	49 (72.1)	56 (82.4)	6
Unno [23] 2010	Japan/2001–2008	42	47	NR	NR	NR	NR	NR	NR	NR	NR	33.2	21.4	NR	NR	6
Shimizu [24] 2010	Japan/1984–2008	88	84	8/39/29/8	10/38/32/8	NR	NR	5	32	25/9	23/2	24.4	14.1	56 (63.6)	58 (69.0)	7
Konstadoulakis [25] 2008	USA/1988–2006	29	20	NR	NR	NR	NR	NR	NR	NR	NR	NR	NR	16 (55.2)	17 (85.0)	7
Kondo [26] 2004	Japan/1999–2002	9	17	0/1/7/1	1/3/8/5	NR	NR	NR	NR	NR	NR	20.8	NR	NR	NR	6
Yamanaka [27] 2001	Japan/1980–1998	11	14	NR	NR	NR	NR	NR	NR	8/9	4/1	NR	NR	11 (91.7)	14 (87.5)	7

*Abbreviations*: *LH* lift-side hepatectomy, *RH* right-side hepatectomy, *EBD* endoscopic biliary drainage, *PBD* percutaneous biliary drainage, *NOS* Newcastle–Ottawa Scale, *NR* not reported in the text

### Survival outcomes

**Fig. 2:**
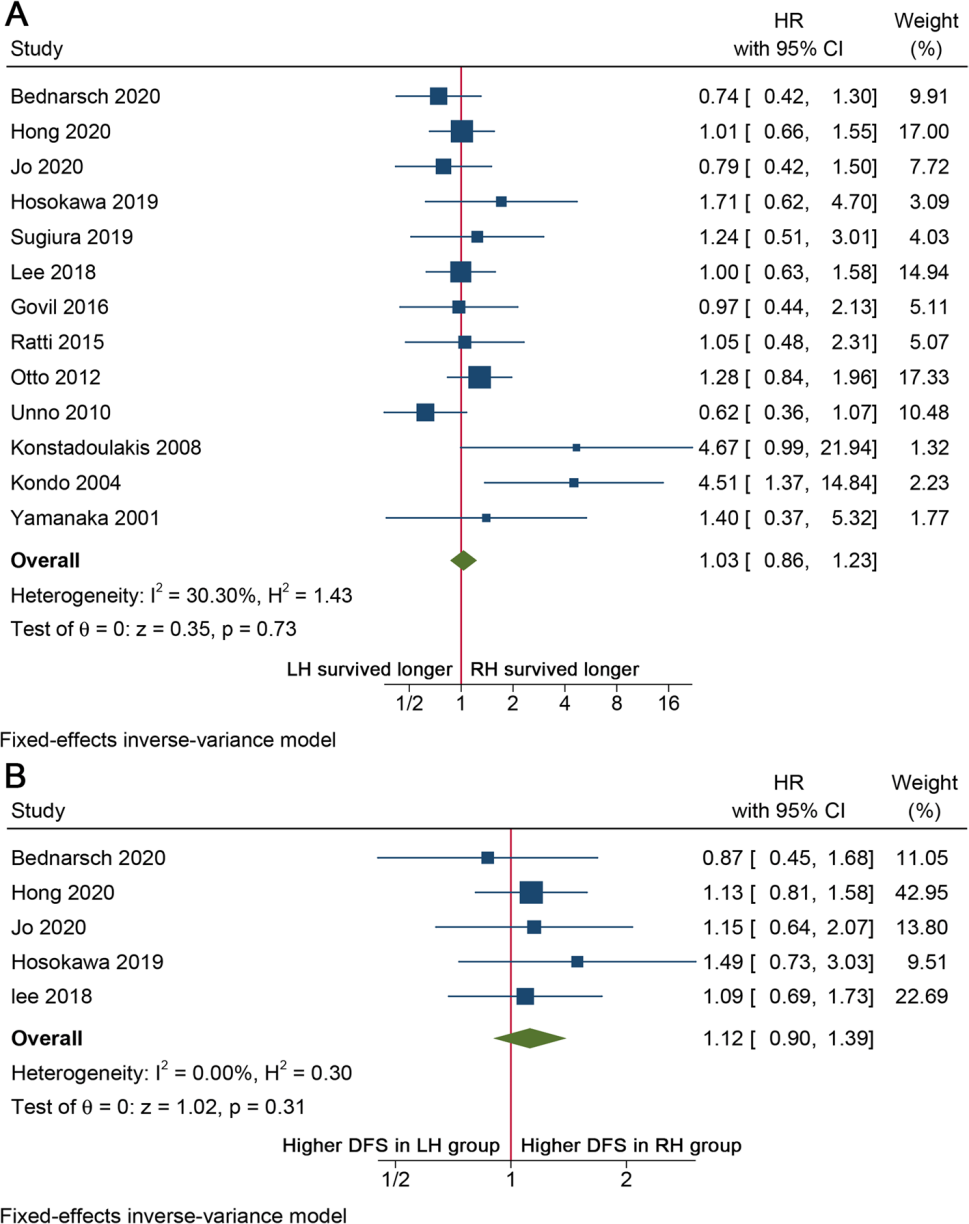
Forest plots for **A** OS and **B** DFS of patients with pCCA between LH and RH

**Fig. 3:**
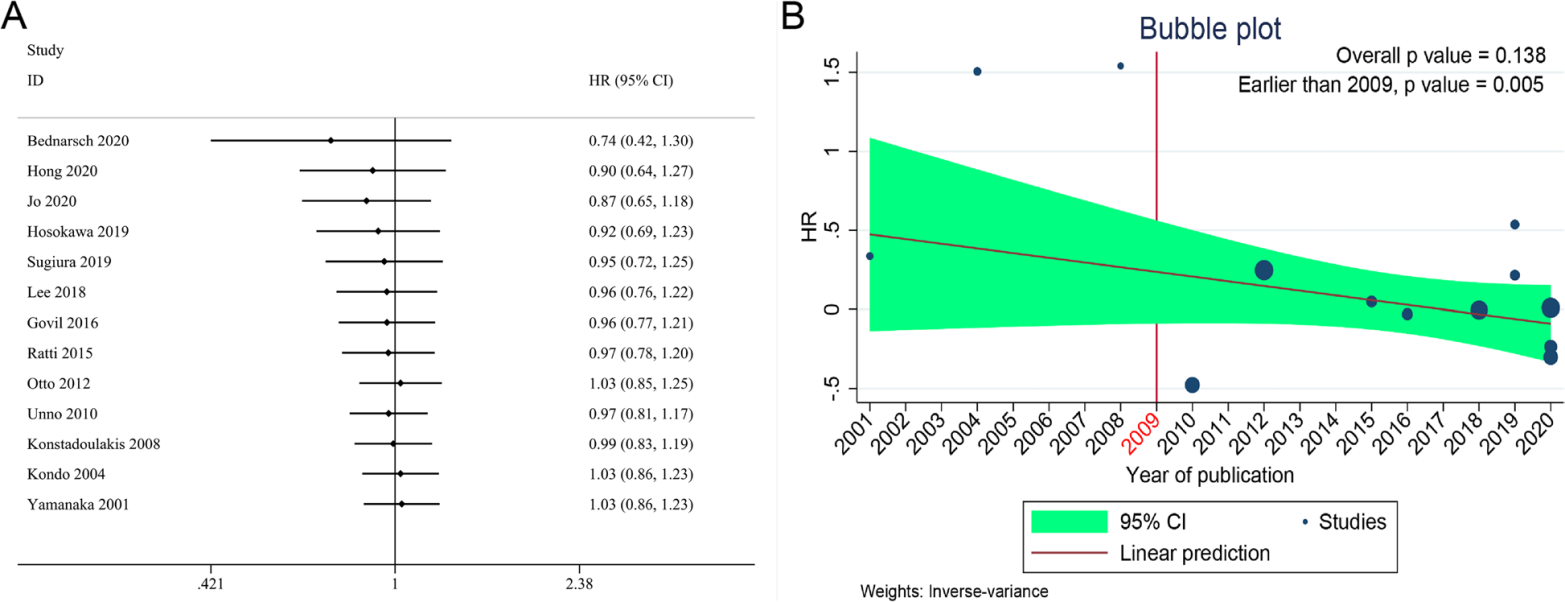
Forest plot of **A** time cumulative meta-analysis and bubble plot of **B** meta-regression according to the year of publication

Thirteen cohort studies [14–23, 25–27] comprising 900 patients reported data of OS, and HR could be either directly extracted from 2 studies [21, 22] or calculated from K-M curve by using the method described above from another 11 studies [14–20, 23, 25–27]. The fixed-effects model was used and pooled HR reveled that there was no significant difference between LH and RH in OS (HR = 1.03, 95%CI 0.86–1.23, *I*^2^ = 30.3%, *P* = 0.73, Fig. 2A). By subsequent cumulative meta-analysis of publication year, the pooled HR was found to cross the invalidation line (HR = 1) at around year 2009 (Fig. 3). Therefore, a subgroup analysis using boundary year of publication as 2009 was conducted, and three studies published earlier than 2009 [25–27] showed a better OS in the RH group than in the LH group (HR = 3.08, 95%CI 1.43–6.66, *I*^2^ = 0.5%, *P* = 0.004), while the other 10 studies published after 2009 showed a comparative OS between LH and RH (HR = 0.97, 95% CI 0.81–1.17, *I*^2^ = 0%, *P* = 0.749, Table S1). The results concerning other subgroup analyses revealed no statistical difference for OS between LH and RH comparing eastern vs. western centers, and studies with higher number of cases (> 100 cases) vs. lower number of cases (< 100 cases) (Table S1). Data of DFS were also reported in 5 studies [14–17, 19] and HR could be extracted with a low heterogeneity (*I*^2^ = 0%), and no statistical difference was found between LH and RH groups (HR = 1.12, 95%CI 0.90–1.39, *P* = 0.31, Fig. 2B).

In stratified analyses, 6 studies [15–17, 19, 22, 25] reported 1-year survival rate, 9 studies [14–19, 21, 24, 25] reported 3-year survival rate, and 9 studies [14–16, 18, 19, 21, 22, 24, 25] reported 5-year survival rate. Pooled OR revealed that LH and RH exhibited comparable 1-year (OR = 1.01, 95%CI 0.68–1.50, *I*^2^ = 49.9%, *P* = 0.96), 3-year (OR = 0.86, 95%CI 0.65–1.14, *I*^2^ = 0%, *P* = 0.30), and 5-year survival (OR = 0.76, 95%CI 0.57–1.01, *I*^2^ = 0%, *P* = 0.06) in pCCA patients (Fig. S1). Similar results were observed in 1-year, 3-year, and 5-year DFS (1-year OR = 0.99, 95%CI 0.64–1.52, *I*^2^ = 0%, *P* = 0.95 [15–17, 19]; 3-year OR = 0.68, 95%CI 0.46–0.99, *I*^2^ = 27.3%, *P* = 0.05 [14–17, 19]; 5-year OR = 0.83, 95%CI 0.50–1.37, *I*^2^ = 0%, *P* = 0.46 [15, 16, 19], Fig. S2). Subgroup analysis showed a better 5-year survival rate of RH group in western centers than in eastern centers, and no statistic difference was observed in other subgroup analyses concerning year of publication and case numbers (Table S1).

### Preoperative biliary drainage and PVE

Preoperative total bilirubin levels were reported in 8 studies [14–17, 19, 21, 24, 27] with no statistical difference between LH and RH groups, although a random-effects model was used (WMD =  − 0.38, 95%CI − 1.42–0.66, *I*^2^ = 90.63%, *P* = 0.47, Fig. S3A). Due to various approaches of biliary drainage were used among studies, data of preoperative biliary drainage could only be aggregated from 5 studies [15, 16, 19–21], and results did not reveal statistical difference between LH and RH groups (RR = 0.91, 95%CI 0.81–1.02, *I*^2^ = 0%, *P* = 0.10, Fig. S3B). PVE was reported in most studies [14–16, 18–22, 24], and data demonstrated that it was broadly performed to increase FLR volume in RH group (RR = 0.07, 95%CI 0.04–0.12, *I*^2^ = 0%, *P* < 0.01, Fig. S3C). Subgroup analysis did not change the final results.

### Operative analyses

**Fig. 4:**
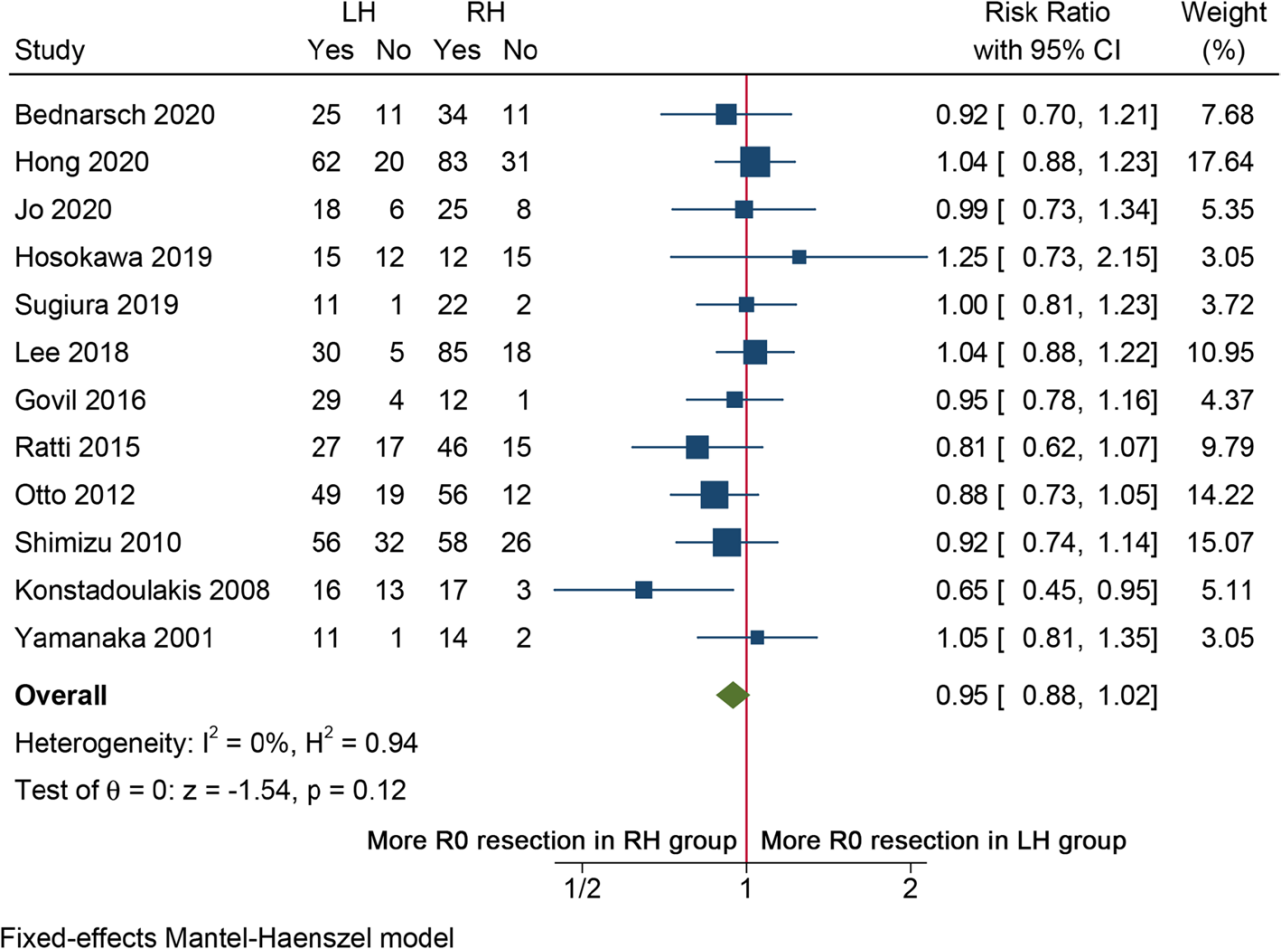
Forest plot of R0 resection rate between LH and RH for pCCA patients

**Fig. 5:**
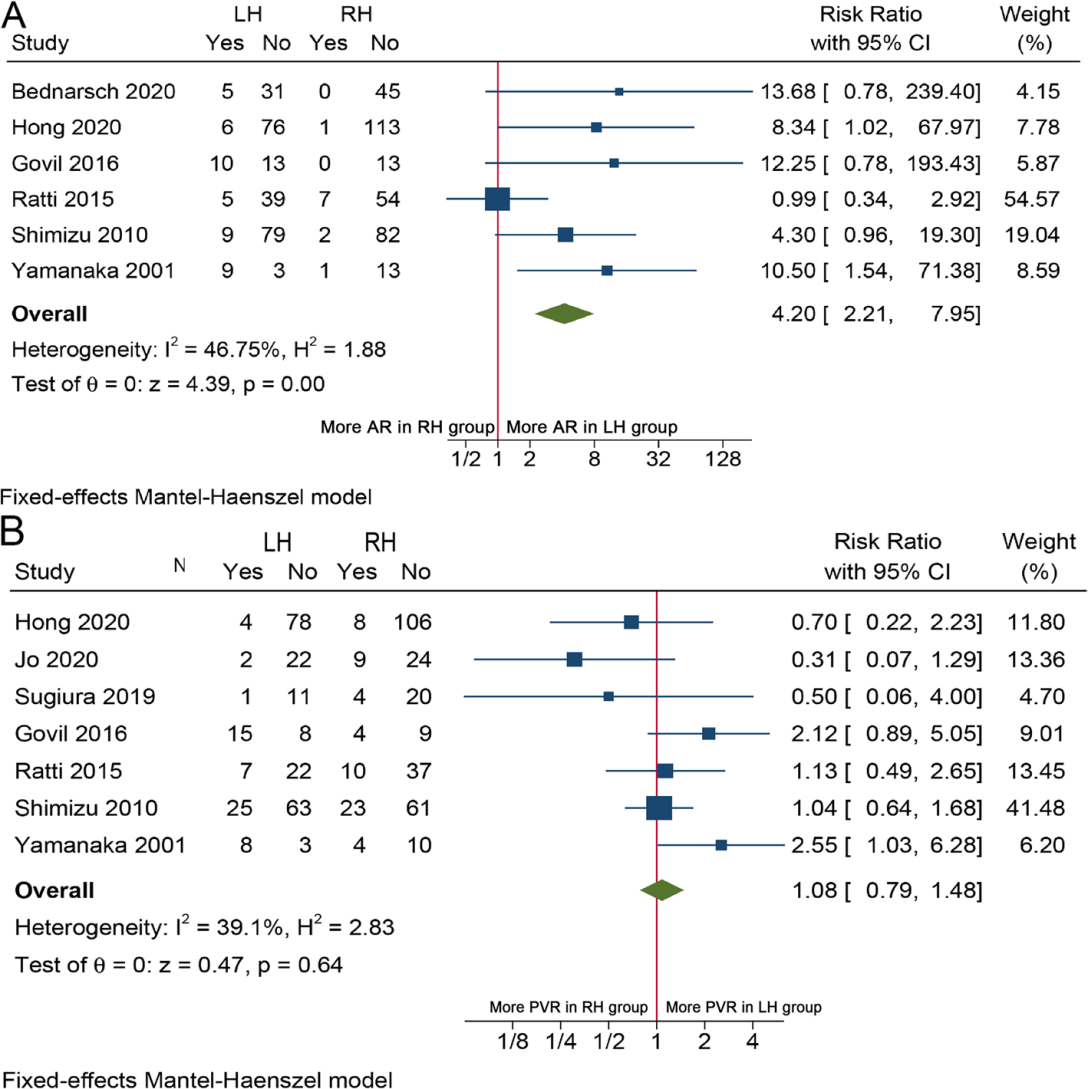
Forest plots of **A** AR and **B** PVR between LH and RH for pCCA patients

A total of 12 studies [14–22, 24, 25, 27] involving 957 patients provided information on R0 resection rate of two different surgical methods. The pooled RR showed that R0 resection rate was comparable between LH and RH (RR = 0.95, 95%CI 0.88–1.02, *I*^2^ = 0%, *P* = 0.12, Fig. 4). As about arterial resection and reconstruction (AR), pooled RR from 6 studies [14, 15, 20, 21, 24, 27] indicated that the rate of AR in the LH group was significantly much higher than in the RH group by fixed-effects model (RR = 4.20, 95%CI 2.21–7.95, *I*^2^ = 46.75%, *P* < 0.01, Fig. 5A). While considering portal vein resection (PVR), data available from 8 studies [14–16, 18, 20, 21, 24, 27] showed no statistical difference between the 2 groups (RR = 1.08, 95%CI 0.79–1.48, *I*^2^ = 39.1%, *P* = 0.64, Fig. 5B). Operative time was reported in 10 studies [14–21, 24, 27], and the results showed that LH usually took longer time than RH (WMD = 31.65, 95%CI 3.77–59.52, *P* = 0.03, Fig. S4A) by random-effect model as significant heterogeneity (*I*^2^ = 66.02%) was observed. However, meta-analysis from 5 studies [15, 16, 19, 21, 24] did not observe statistical difference in intraoperative blood loss (WMD =  − 15.10, 95%CI − 85.62–115.82, *I*^2^ = 32.1%, *P* = 0.77, Fig. S4B) and transfusion rate (RR = 1.16, 95%CI 0.99–1.37, *I*^2^ = 0%, *P* = 0.07, Fig. S4C) between the two surgical approaches. Subgroup analyses demonstrated that LH was significantly associated with higher rate of AR in eastern centers and lower rate of R0 resection in western centers (Table S1, S2). The LH group also has longer operation time in western centers and higher number of cases (> 100 cases) centers.

### Postoperative complications and mortality

Only four studies [14, 15, 21, 24] incorporating 554 patients reported the overall postoperative complications. RH was significantly correlated with higher rate of overall complications (RR = 0.82, 95%CI 0.71–0.96, *I*^2^ = 13.92%, *P* = 0.01, Fig. S5A) by fixed-effects model. Another 6 studies [14, 16–18, 20, 21] enrolling 365 patients reported major complications (Clavien-Dindo III-V) showed no statistical difference between LH and RH (RR = 0.82, 95%CI 0.65–1.03, *I*^2^ = 34.37%, *P* = 0.09, Fig. S5B). Furthermore, we focused on occurrence of the most serious complications after hepatectomy which were PHLF and postoperative bile leakage. Six studies [15, 16, 18, 20, 21, 27] involving 455 patients reported the occurrence of PHLF to be higher in the RH group than in the LH group with a significant difference (RR = 0.26, 95%CI 0.12–0.56, *I*^2^ = 0%, *P* < 0.01, Fig. S5C), while data from 6 other studies [15, 18, 20, 21, 24, 27] by fixed-effect model showed that patients received LH were more likely to have postoperative bile leakage than RH (RR = 1.91, 95%CI 1.17–3.11, *I*^2^ = 0%, *P* = 0.01, Fig. S5D).

Subgroup analyses between different regions and enrolled case numbers were conducted as shown in Table S2. Data suggested that in western centers, RH was notably associated with more overall complications and major complications (overall complications:* P* < 0.01, major complications: *P* = 0.02). In centers with higher number of cases (> 100 cases), LH was significantly associated with more bile leakage than RH (*P* < 0.01), but no significant difference in the incidence of PHLF was observed. In centers with lower number of cases (< 100 cases), RH was associated with higher rate of PHLF than LH (*P* < 0.01), and no statistical difference for postoperative bile leakage was observed.

Ten studies [14–17, 19–22, 24, 27] including 813 patients reported the postoperative mortality, with three of them [16, 17, 21] reported 90-day mortality after operation and seven of them [14, 15, 19, 20, 22, 24, 27] reported whole in-hospital mortality (or perioperative mortality). The results of meta-analysis showed that LH was significantly associated with lower rate of both overall postoperative mortality (RR = 0.52, 95%CI 0.31–0.86, *I*^2^ = 0%, *P* = 0.01, Fig. S6A) and in-hospital mortality (or perioperative mortality) (RR = 0.42, 95%CI 0.23–0.75, *I*^2^ = 0%, *P* < 0.01, Fig. S6B).

By subgroup analyses concerning mortality (Table S2), LH was associated with reduced overall mortality in western centers (*P* = 0.02) and reduced in-hospital mortality in eastern centers (*P* = 0.03). While in centers with higher number of cases (> 100 cases), LH was observed to have notably reduced overall and in-hospital mortalities (overall mortality:* P* < 0.01, in-hospital mortality:* P* < 0.01). No statistical differences were observed in other subgroup analyses.

### Publication bias

There was no significant publication bias for both OS (Egger’s test, *P* = 0.052) and R0 resection rate (Egger’s test, *P* = 0.484) among the included studies, and the funnel plot was almost symmetrical (Fig. S7).

## Discussion

Major liver resections including RH (right-sided hepatectomy, i.e., right hemi-hepatectomy and right trisectionectomy) and LH (left-sided hepatectomy, i.e., left hemi-hepatectomy and left trisectionectomy) with caudate lobectomy and radical lymph node dissection are regarded as the standard surgery for curative pCCA resection [5, 6]. This meta-analysis focused on comparative study between LH and RH, hoping to provide some clinical evidence for decision making on surgical strategies. Our results showed there was no significant difference between LH and RH in terms of pooled HR for OS, DFS, R0 resection rate, 1-, 3-, and 5-year survival rates. Some other perioperative outcomes between the 2 groups were statistically different, as the RH group exhibited more use of PVE and higher rate of postoperative overall complications, PHLF, operative, or in-hospital mortality, while the LH group was associated with more use of AR and longer operative time.

Traditionally, Bismuth-Corlette classification is widely used for preoperative evaluation of tumor location and range of biliary infiltration, facilitating surgery choice between LH and RH [7]. Currently, the choice of surgical strategy between RH and LH is determined by not only tumor locations, but also vascular invasion and FLR evaluation [14–16]. For pCCA, it is sometimes difficult to achieve R0 resection due to tumor biology and anatomical complexity [28, 29]. Indeed, the distance from the primary bile duct bifurcation to the sectional branch is much shorter in the right side of the liver than in the left, and there are many anatomical variations in the right sectional bile ducts [30]. And also, the right hepatic artery (RHA) runs closely behind the biliary confluence which makes it more susceptible to tumor invasion. Furthermore, LH usually leaves more ductal remnant stumps requiring more bilioenteric anastomoses which means higher risk of bile leakage. Considering all these together that RH may hold anatomical advantages over LH for radicality, most surgeons prefer RH than LH, as en bloc resection of the RHA and surrounding tissues could be performed [31–33]. Konstadoulakis et al. [25] found that patients with RH had a significantly lower rate of tumor-positive margin than those with LH, while Kondo et al*.* [26] and Yamanaka et al. [27] demonstrated in their studies that the survival of patients with RH was better than those with LH. Thus, RH is more favored than LH by most surgeons for the treatment of pCCA to obtain better radicality and survival [34]. Not until 2010, Nagino et al. [35] reported LH combined with vascular reconstruction in 49 out of 50 pCCA patients with an acceptable 2% operative mortality and 30% 5-year survival, did LH become widely performed for pCCA. Since then, more and more studies have demonstrated no significant differences between LH and RH in terms of radicality and long-term survival, as 5-year survival of pCCA patients significantly improved to 30–45% [14, 36, 37].

Concerning survival analysis, we found no statistical difference in OS and DFS between LH and RH by meta-analysis. Although the pooled HR showed low heterogeneity in OS (*I*^2^ = 30.3%) and DFS (*I*^2^ = 0–27.3%), we still performed subgroup analyses and meta-regressions, trying to find out the source of making no survival difference between LH and RH. Data revealed that except for the year of publication, neither the study region (eastern centers or western centers) nor the number of surgical cases (> 100 cases or < 100 cases) reduced heterogeneity or changed the results. Through cumulative meta-analysis of the publication year, we found that pooled HR crossed the invalidation line at around year 2009. Subsequently, we subdivided the data by year of 2009 and revealed significantly reduced heterogeneity with a better OS for RH before 2009 (*P* = 0.004) and a comparative OS for LH since after 2009 (*P* = 0.749). We speculate that this may be related to improvements of surgical techniques especially employment of AR (see below) in LH as experiences accumulate.

R0 resection not only needs to ensure a negative margin of bile duct, but also requires clearance of tumor invaded vasculatures and surrounding tissues. The procedure of vascular resection and reconstruction (VR) plays an important role in curative resection of pCCA. Generally, PVR is more frequently performed than HAR and accepted to contribute to increased resectability and improved prognosis [16]. In a systematic review and meta-analysis of the use of PVR in the treatment of pCCA, Chen et al. concluded that combined PVR was safe and feasible in the treatment of pCCA, and PVR could increase resectability and benefit the overall survival in certain patients with grossly involved portal vein [38]. In our meta-analysis, PVR is almost equally performed in both LH and RH with comparable morbidity and mortality which agrees with previous reports. But for HAR, the issue becomes more complicated and controversial as it is technically demanding and challenging. It is not until 2010, Nagino et al. [35] reported their experiences of combined PVR and HAR for pCCA patients with excellent outcomes, has HAR been advocated and few studies been reported [16, 39]. In our meta-analysis, we found that HAR is dominantly performed with LH and is consistent with our conventional understanding. As mentioned above, RH tends to be more sufficient on radicality as RHA could be removed during operation, while LH always requires HAR to achieve the same negative resection margin [33, 40]. AR has been found to be associated with poorer oncological outcomes and higher postoperative mortality, which may be explained by the fact that more patients die from revascularization complications [35, 40]. Through our meta-analysis, we found that although the LH group employed more use of HAR, there was no statistical difference in OS and R0 resection rate compared to the RH group. A further subgroup analysis revealed that HAR was more frequently employed in the LH group in eastern centers with higher R0 resection rate and better 5-year survival compared to in western centers. Nevertheless, higher in-hospital or perioperative mortalities in eastern centers where more HAR was employed in spite of its safety and feasibility [41]. Due to lack of high-quality randomized control trials, it still needs further investigation whether AR could increase R0 resection rate or long-term survival of LH patients.

Our meta-analysis revealed that the RH group was associated with higher rate of overall complications, PHLF, and postoperative mortality, which was also consistent with our conventional understanding. However, no statistical difference was observed in major complications (Clavien-Dindo ≥ III grade). One meta-analysis on incidence of complications and mortality after major hepatectomies in pCCA patients by Franken et al*.* [42] showed a better outcome of mortality in eastern centers than in western centers. The authors believed that surgeons in eastern centers had more experiences in operation because of higher prevalence of pCCA in Asian countries. Data of our subgroup analysis also agreed that the RH group in western centers was associated with higher rate of overall and major complications, as well as peri-operative mortality. However, when reporting mortality, the standards used among studies were different which may affect the power of statistics. Next, we conducted subgroup analysis by different case numbers to see if patient volume may affect the results. Data showed that the RH group of centers with more than 100 cases had a higher mortality rate, which could be explained that high-volume centers tend to admit more complicated patients, although they are considered to have more experience [42]. We also found that centers with lower number of surgical cases had more PHLF in the RH group, which could be explained by the lack of experience in perioperative management, such as application of PVE and biliary drainage.

Indeed, PHLF caused by insufficient FLR is the most fetal complication after major hepatectomy, with a mortality rate of 52–68% [43]. Most studies agree that preoperative PVE and biliary drainage are effective in increasing FLR and improving liver function, thereby reducing PHLF. However, our review of 5 studies did not find significant differences in preoperative biliary drainage between the LH and RH groups [15, 16, 19–21]. Another 9 studies supported more use of PVE in the RH group than in the LH group with statistical difference [14–16, 18–22, 24]. But one point should not be neglected that either PVE or biliary drainage will cause delay for operation, and the average time lag was estimated to be about 25 days in previous studies [14, 15], which could witness potential risk of tumor progression. Thus, it seems like LH exhibits some extent of advantage as it exempts use of PVE and does not cause delay for surgery.

There were some limitations in this meta-analysis. Firstly, all the included studies were retrospective cohort studies, further high-quality randomized control trials should be designed and included for future investigations. Secondly, although most included studies provided information about Bismuth classification which is important indicator for selection of LH or RH for pCCA patients, we could not extract detailed data of each type (especially type I/II/IV) for subgroup analyses, which may affect the accuracy of the overall results.

According to our meta-analyses, LH and RH have comparable oncological effects on curative resection for pCCA patients. Although LH is not inferior to RH in DFS and OS, it requires more arterial reconstruction which is technically demanding and should be performed by experienced surgeons in high-volume centers. Selectin of surgical strategy between LH and RH should be based on not only tumor location (Bismuth classification) but also vascular involvement and FLR.

## Supplementary Information

**Additional file 1: Table S1.** Subgroup analyses of major survival outcomes. **Table S2. **Subgroup analyses of peri-operative outcomes.

**Additional file 2:** **Fig. S1.** Forest plot for the 1-year, 3-year and 5-year survival rates of patients with Hilar cholangiocarcinoma (HCCA) between left-side hepatectomy (LH) and right-side hepatectomy (RH). A 1-year survival rate. B 3-year survival rate. C 5-year survival rate. **Fig. S2.** Forest plot for the 1-year, 3-year and 5-year disease-free survival rate of patients with Hilar cholangiocarcinoma (HCCA) between left-side hepatectomy (LH) and right-side hepatectomy (RH). A 1-year disease-free survival rate. B 3-year disease-free survival rate. C 5-year disease-free survival rate. **Fig. S3.** Forest plots of A) preoperative total bilirubin levels, B) preoperative biliary drainage and C) portal vein embolization (PVE) between left-side hepatectomy (LH) and right-side hepatectomy (RH). **Fig. S4.** Forest plots of A) operation time, B) postoperative bile leakage, and C) intraoperative transfusion rates between left-side hepatectomy (LH) and right-side hepatectomy (RH). **Fig. S5.** Forest plots of A) overall postoperative morbidity, B) major postoperative morbidity, C) post-hepatectomy liver failure (PHLF), and postoperative bile leakage between left-side hepatectomy (LH) and right-side hepatectomy (RH). **Fig. S6.** Forest plots of A) overall postoperative mortality and B) in-hospital mortality (or perioperative motility) between left-side hepatectomy (LH) and right-side hepatectomy (RH). **Fig. S7.** Funnel plot of A) overall survival and B) R0 resection.

## Data Availability

The data analyzed during the current study are available from the corresponding author on reasonable request.
