# Supplementary material for: Comparative study on left-sided versus right-sided hepatectomy for resectable peri-hilar cholangiocarcinoma: a systematic review and meta-analysis
Source: World J Surg Oncol. 2023 May 18;21:153. doi: 10.1186/s12957-023-03037-2 (PMC10193683; doi:10.1186/s12957-023-03037-2)
Supplement: Supplementary file 1 — Additional file 1: Table S1. Subgroup analyses of major survival outcomes. Table S2. Subgroup analyses of peri-operative outcomes. [file 12957_2023_3037_MOESM1_ESM.docx]

Supplementary_Table_1. Subgroup analyses of major survival outcomes.

| Variable | Subgroup | OS | 1-year survival | 3-year survival | 5-year survival | DFS | 1-year DFS | 3-year DFS | 5-year DFS | R0 resection | Artery resection |
| --- | --- | --- | --- | --- | --- | --- | --- | --- | --- | --- | --- |
| Year of publication | >2009 | HR=0.97,  95%CI 0.81–1.17, *P*=0.75, n=10 | n=5 | n=7 | n=7 | n=5 | n=4 | n=5 | n=3 | RR=0.96,  95%CI 0.89–1.03, *P*=0.27, n=10 | n=5 |
|  | <2009 | HR=3.08,  95%CI 1.43–6.66, *P*<0.01, n=3 | n=1 | n=1 | n=1 | n=0 | n=0 | n=0 | n=0 | RR=0.84,  95%CI 0.49–1.43, *P*=0.52, n=2 | n=1 |
| Region | Eastern | HR=0.99,  95%CI 0.80–1.24, *P*=0.96, n=9 | OR=1.07,  95%CI 0.45–2.53, *P*=0.87, n=4 | OR=0.86,  95%CI 0.61–1.21, *P*=0.39, n=6 | OR=0.92,  95%CI 0.63–1.34, *P*=0.65, n=5 | n=4 | n=4 | n=4 | n=3 | RR=0.98,  95%CI 0.90–1.07, *P*=0.67, n=9 | RR=7.48, 95%CI 2.86–19.58, *P*<0.01, n=4 |
|  | Western | HR=1.11,  95%CI 0.82–1.51, *P*=0.50, n=4 | OR=0.68,  95%CI 0.27–1.71, *P*=0.41, n=2 | OR=0.79,  95%CI 0.34–1.84, *P*=0.59, n=3 | OR=0.58,  95%CI 0.37–0.91, *P*=0.02, n=4 | n=1 | n=0 | n=1 | n=0 | RR=0.87,  95%CI 0.76–0.99, *P*=0.04, n=3 | RR=2.71, 95%CI 0.19–38.82, *P*=0.46, n=2 |
| Patients | >100 cases | HR=0.99,  95%CI 0.80–1.24, *P*=0.96, n=5 | OR=1.09,  95%CI 0.53–2.22, *P*=0.81, n=3 | OR=0.89,  95%CI 0.63–1.27, *P*=0.53, n=4 | OR=0.83,  95%CI 0.59–1.16, *P*=0.30, n=5 | HR=1.12,  95%CI 0.85–1.46, P=0.43, n=2 | OR=1.17,  95%CI 0.71–1.95, P=0.54, n=2 | OR=0.67,  95%CI 0.41–1.11, P=0.11, n=2 | n=2 | RR=0.95,  95%CI 0.87–1.03, *P*=0.21, n=5 | RR=2.47, 95%CI 1.17–5.22, *P*=0.02, n=3 |
|  | <100 cases | HR=1.11,  95%CI 0.82–1.49, *P*=0.51, n=8 | OR=0.67,  95%CI 0.19–2.33, *P*=0.52, n=3 | OR=0.80,  95%CI 0.49–1.30, *P*=0.36, n=5 | OR=0.27,  95%CI 0.34–1.04, *P*=0.07, n=4 | HR=1.13,  95%CI 0.78–1.64, P=0.52, n=3 | OR=0.59,  95%CI 0.25–1.40, P=0.23, n=2 | OR=0.62,  95%CI 0.22–1.69, P=0.35, n=3 | n=1 | RR=0.95,  95%CI 0.84–1.07, *P*=0.36, n=7 | RR=11.76, 95%CI 2.90–47.73, *P*<0.01, n=3 |

Data are presented as HR (95% CI), OR (95% CI) or RR (95% CI); *P* value; number of included studies (n)

Abbreviations: OS: overall survival, DFS: disease free survival,

Supplementary_Table_2. Subgroup analyses of peri-operative outcomes.

| Variable | Subgroup | Preoperative bilirubin | Preoperative biliary drainage | PVE | PVR | Operation time | Intraoperative blood loss | Transfusion rate | Overall complications | Major complications | PHLF | Bile leakage | Mortality | In-Op mortality |
| --- | --- | --- | --- | --- | --- | --- | --- | --- | --- | --- | --- | --- | --- | --- |
| Year of publication | >2009 | n=7 | n=5 | n=8 | n=6 | n=9 | n=6 | n=5 | n=4 | n=6 | n=5 | n=5 | n=9 | n=7 |
|  | <2009 | n=1 | n=0 | n=0 | n=1 | n=1 | n=1 | n=0 | n=0 | n=0 | n=1 | n=1 | n=1 | n=1 |
| Region | Eastern | WMD=-0.45, 95%CI -1.07–0.18, P=0.10, n=6 | n=4 | RR=0.09, 95%CI 0.04–0.18, P<0.01, n=6 | n=6 | WMD=30.65, 95%CI -5.01–66.31, P=0.09, n=8 | n=6 | n=4 | RR=0.92, 95%CI 0.74–1.14, *P*=0.43, n=2 | RR=1.00, 95%CI 0.76–1.33, P=0.98, n=4 | n=5 | n=5 | RR=0.60, 95%CI 0.31–1.14, *P*=0.12, n=7 | RR=0.42, 95%CI 0.19–0.091, P=0.03, n=5 |
|  | Western | WMD=-0.59, 95%CI -5.19–4.02, P=0.80, n=2 | n=1 | RR=0.03, 95%CI 0.01–0.13, P<0.01, n=3 | n=1 | WMD=47.83, 95%CI 14.19–81.46, P<0.01, n=2 | n=1 | n=1 | RR=0.7, 95%CI 0.57–0.87, *P*<0.01, n=2 | RR=0.62, 95%CI 0.42–0.91, P=0.02, n=2 | n=1 | n=1 | RR=0.39, 95%CI 0.17–0.88, *P*=0.02, n=3 | RR=0.42, 95%CI 0.17–1.02, P=0.05, n=2 |
| Patients | >100 cases | WMD=-0.84, 95%CI -2.30–0.63, P=0.26, n=4 | RR=0.89, 95%CI 0.78–1.02, P=0.09, n=3 | RR=0.08, 95%CI 0.04–0.16, P<0.01, n=5 | RR=1.00, 95%CI 0.67–1.48, P=0.99, n=3 | WMD=19.97, 95%CI 0.40–39.54, P=0.046, n=4 | WMD=55.02, 95%CI -55.21–165.26, P=0.33, n=3 | n=4 | n=3 | n=1 | RR=0.38, 95%CI 0.11–1.32, *P*=0.13, n=2 | RR=2.10, 95%CI 1.20–3.69, P<0.01, n=3 | RR=0.32, 95%CI 0.16–0.65, *P*<0.01, n=5 | RR=0.33, 95%CI 0.16–0.69, P<0.01, n=4 |
|  | <100 cases | WMD=0.01, 95%CI -1.25–1.26, P=0.99, n=4 | RR=0.97, 95%CI 0.78–1.21, P=0.81, n=2 | RR=0.03, 95%CI 0.01–0.15, P<0.01, n=3 | RR=1.19, 95%CI 0.45–3.12, P=0.73, n=4 | WMD=49.57, 95%CI -7.56–106.70, P=0.09, n=6 | WMD=-186.75, 95%CI -434.62–61.11, P=0.14, n=4 | n=1 | n=1 | n=5 | RR=0.21, 95%CI 0.08–0.56, *P*<0.01, n=4 | RR=1.40, 95%CI 0.52–3.80, P=0.51, n=3 | RR=1.01, 95%CI 0.46–2.25, *P*=0.97, n=5 | RR=0.70, 95%CI 0.26–1.94, P=0.50, n=3 |

Data are presented as WMD (95%CI), OR (95%CI) or RR (95%CI); P value; number of included studies (n)

Abbreviations: PVE: Selective portal vein embolization, PVR: Portal vein resection, PHLF: postoperative liver failure, In-Op Mortality: in-hospital motility or perioperative motility,
