# Supplementary figures and images for: Comparative study on left-sided versus right-sided hepatectomy for resectable peri-hilar cholangiocarcinoma: a systematic review and meta-analysis
Source: World J Surg Oncol. 2023 May 18;21:153. doi: 10.1186/s12957-023-03037-2 (PMC10193683; doi:10.1186/s12957-023-03037-2)

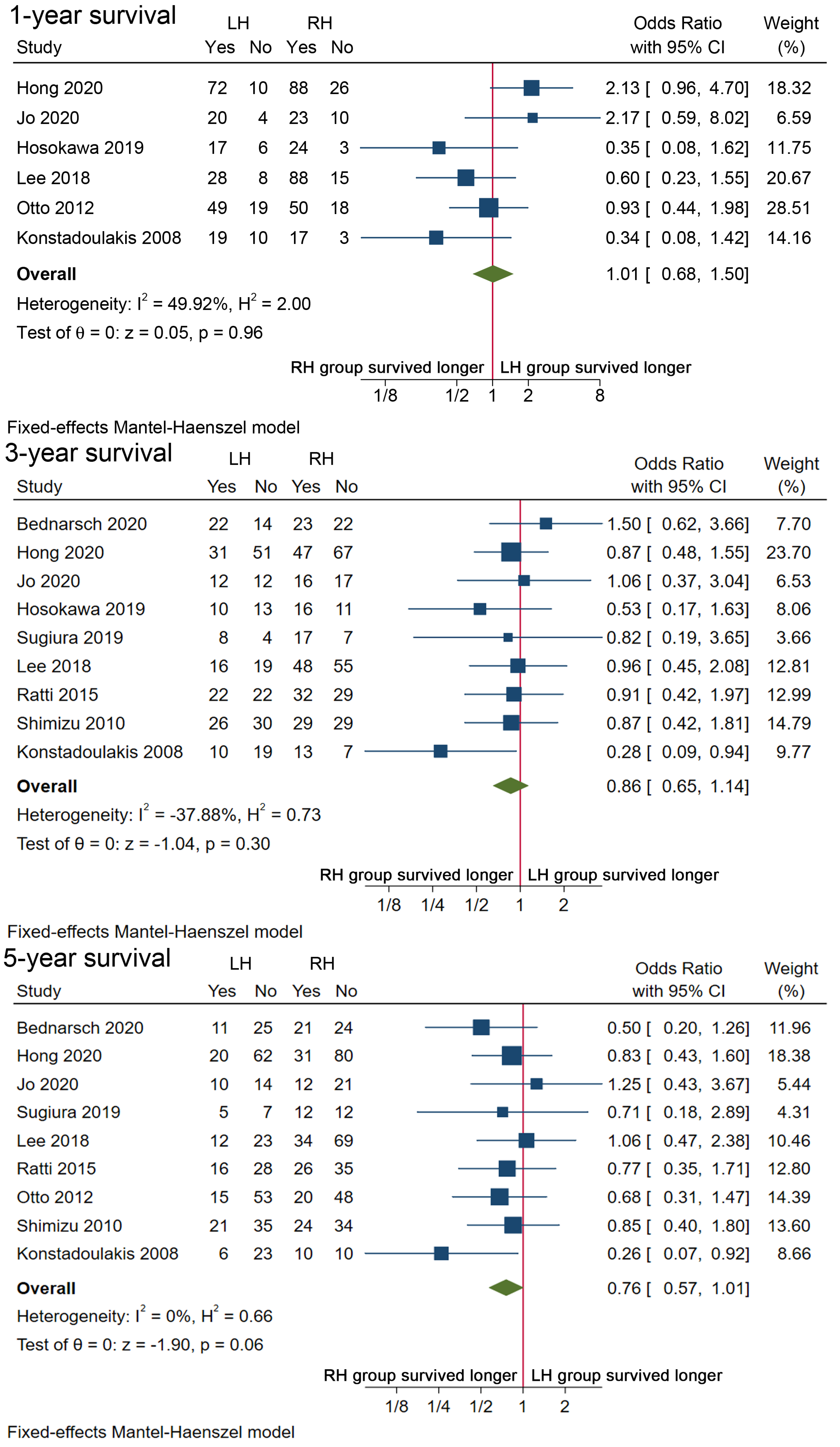

Supplement: Supplementary file 2 — Additional file 2: Fig. S1. Forest plot for the 1-year, 3-year and 5-year survival rates of patients with Hilar cholangiocarcinoma (HCCA) between left-side hepatectomy (LH) and right-side hepatectomy (RH). A 1-year survival rate. B 3-year survival rate. C 5-year survival rate. Fig. S2. Forest plot for the 1-year, 3-year and 5-year disease-free survival rate of patients with Hilar cholangiocarcinoma (HCCA) between left-side hepatectomy (LH) and right-side hepatectomy (RH). A 1-year disease-free survival rate. B 3-year disease-free survival rate. C 5-year disease-free survival rate. Fig. S3. Forest plots of A) preoperative total bilirubin levels, B) preoperative biliary drainage and C) portal vein embolization (PVE) between left-side hepatectomy (LH) and right-side hepatectomy (RH). Fig. S4. Forest plots of A) operation time, B) postoperative bile leakage, and C) intraoperative transfusion rates between left-side hepatectomy (LH) and right-side hepatectomy (RH). Fig. S5. Forest plots of A) overall postoperative morbidity, B) major postoperative morbidity, C) post-hepatectomy liver failure (PHLF), and postoperative bile leakage between left-side hepatectomy (LH) and right-side hepatectomy (RH). Fig. S6. Forest plots of A) overall postoperative mortality and B) in-hospital mortality (or perioperative motility) between left-side hepatectomy (LH) and right-side hepatectomy (RH). Fig. S7. Funnel plot of A) overall survival and B) R0 resection. [file 12957_2023_3037_MOESM2_ESM.zip › S1.tif]

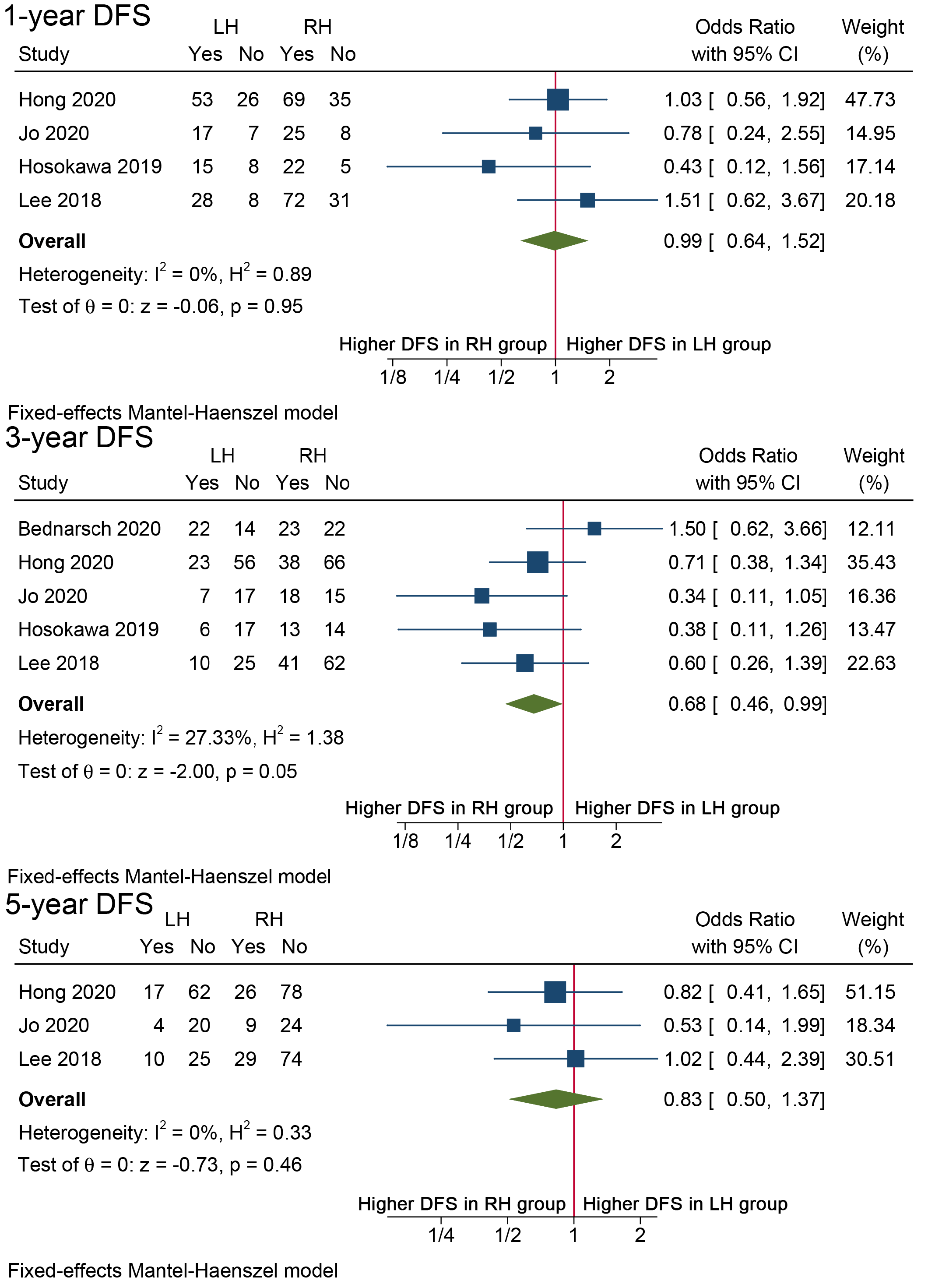

Supplement: Supplementary file 2 — Additional file 2: Fig. S1. Forest plot for the 1-year, 3-year and 5-year survival rates of patients with Hilar cholangiocarcinoma (HCCA) between left-side hepatectomy (LH) and right-side hepatectomy (RH). A 1-year survival rate. B 3-year survival rate. C 5-year survival rate. Fig. S2. Forest plot for the 1-year, 3-year and 5-year disease-free survival rate of patients with Hilar cholangiocarcinoma (HCCA) between left-side hepatectomy (LH) and right-side hepatectomy (RH). A 1-year disease-free survival rate. B 3-year disease-free survival rate. C 5-year disease-free survival rate. Fig. S3. Forest plots of A) preoperative total bilirubin levels, B) preoperative biliary drainage and C) portal vein embolization (PVE) between left-side hepatectomy (LH) and right-side hepatectomy (RH). Fig. S4. Forest plots of A) operation time, B) postoperative bile leakage, and C) intraoperative transfusion rates between left-side hepatectomy (LH) and right-side hepatectomy (RH). Fig. S5. Forest plots of A) overall postoperative morbidity, B) major postoperative morbidity, C) post-hepatectomy liver failure (PHLF), and postoperative bile leakage between left-side hepatectomy (LH) and right-side hepatectomy (RH). Fig. S6. Forest plots of A) overall postoperative mortality and B) in-hospital mortality (or perioperative motility) between left-side hepatectomy (LH) and right-side hepatectomy (RH). Fig. S7. Funnel plot of A) overall survival and B) R0 resection. [file 12957_2023_3037_MOESM2_ESM.zip › S2.tif]

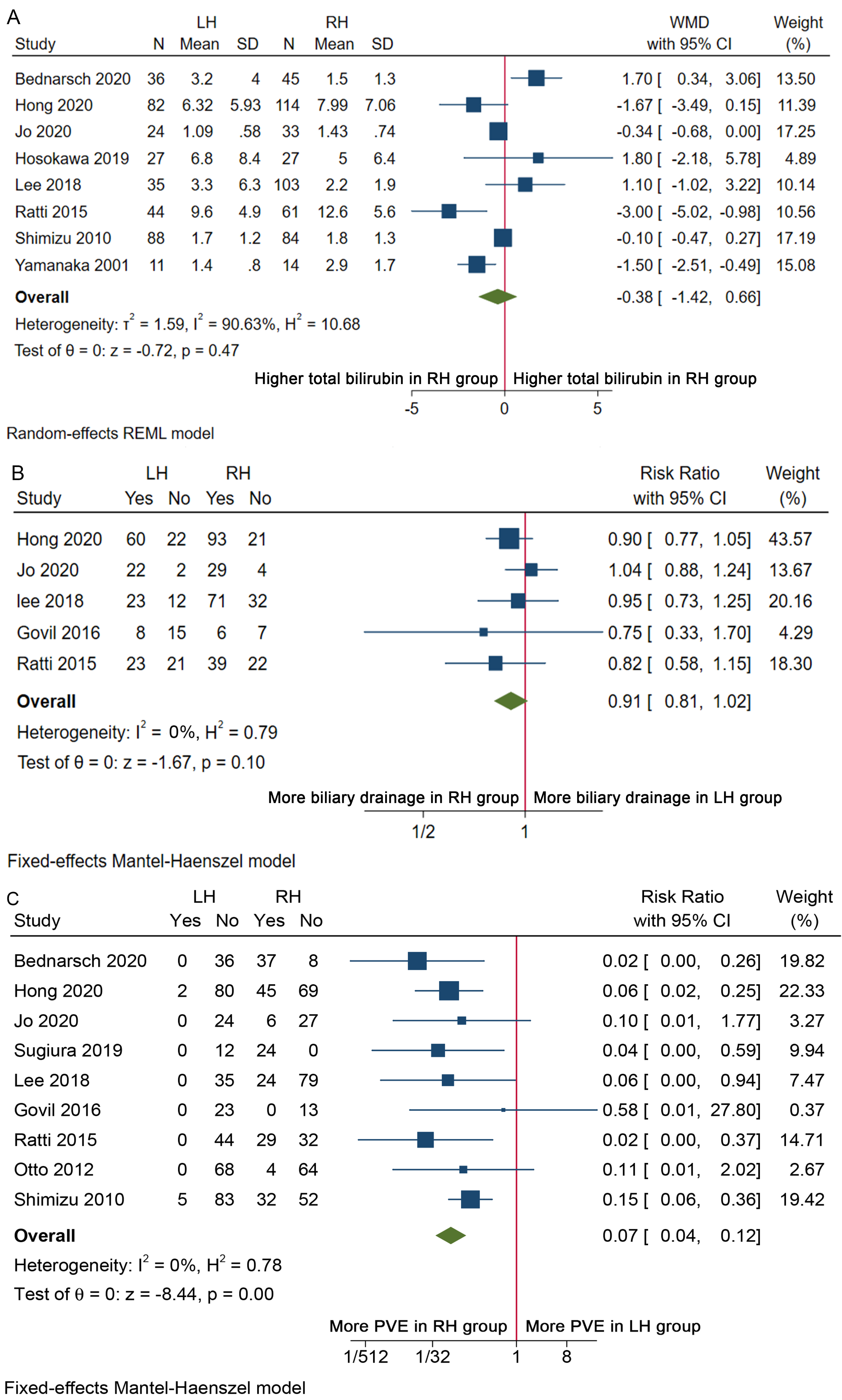

Supplement: Supplementary file 2 — Additional file 2: Fig. S1. Forest plot for the 1-year, 3-year and 5-year survival rates of patients with Hilar cholangiocarcinoma (HCCA) between left-side hepatectomy (LH) and right-side hepatectomy (RH). A 1-year survival rate. B 3-year survival rate. C 5-year survival rate. Fig. S2. Forest plot for the 1-year, 3-year and 5-year disease-free survival rate of patients with Hilar cholangiocarcinoma (HCCA) between left-side hepatectomy (LH) and right-side hepatectomy (RH). A 1-year disease-free survival rate. B 3-year disease-free survival rate. C 5-year disease-free survival rate. Fig. S3. Forest plots of A) preoperative total bilirubin levels, B) preoperative biliary drainage and C) portal vein embolization (PVE) between left-side hepatectomy (LH) and right-side hepatectomy (RH). Fig. S4. Forest plots of A) operation time, B) postoperative bile leakage, and C) intraoperative transfusion rates between left-side hepatectomy (LH) and right-side hepatectomy (RH). Fig. S5. Forest plots of A) overall postoperative morbidity, B) major postoperative morbidity, C) post-hepatectomy liver failure (PHLF), and postoperative bile leakage between left-side hepatectomy (LH) and right-side hepatectomy (RH). Fig. S6. Forest plots of A) overall postoperative mortality and B) in-hospital mortality (or perioperative motility) between left-side hepatectomy (LH) and right-side hepatectomy (RH). Fig. S7. Funnel plot of A) overall survival and B) R0 resection. [file 12957_2023_3037_MOESM2_ESM.zip › S3.tif]

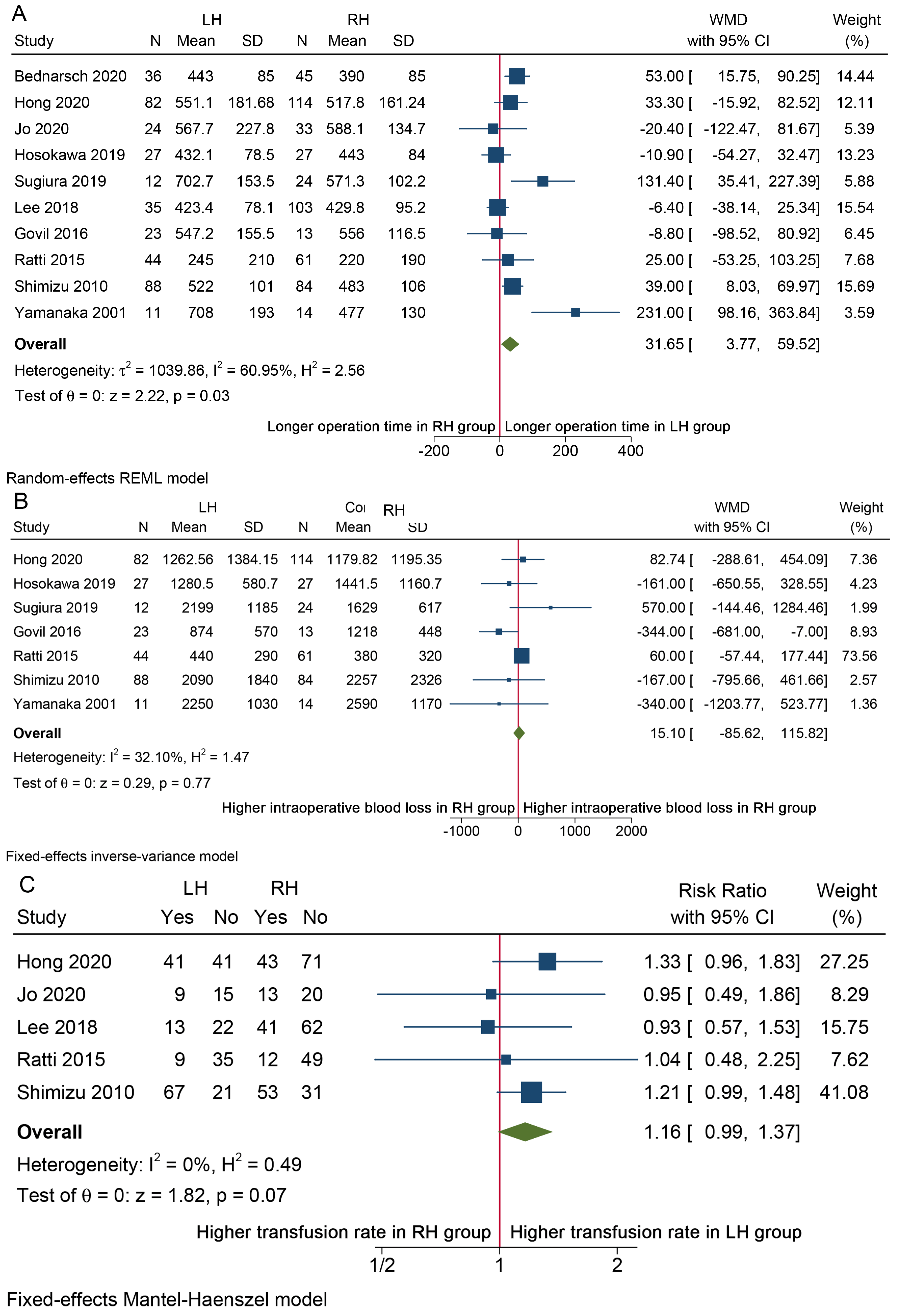

Supplement: Supplementary file 2 — Additional file 2: Fig. S1. Forest plot for the 1-year, 3-year and 5-year survival rates of patients with Hilar cholangiocarcinoma (HCCA) between left-side hepatectomy (LH) and right-side hepatectomy (RH). A 1-year survival rate. B 3-year survival rate. C 5-year survival rate. Fig. S2. Forest plot for the 1-year, 3-year and 5-year disease-free survival rate of patients with Hilar cholangiocarcinoma (HCCA) between left-side hepatectomy (LH) and right-side hepatectomy (RH). A 1-year disease-free survival rate. B 3-year disease-free survival rate. C 5-year disease-free survival rate. Fig. S3. Forest plots of A) preoperative total bilirubin levels, B) preoperative biliary drainage and C) portal vein embolization (PVE) between left-side hepatectomy (LH) and right-side hepatectomy (RH). Fig. S4. Forest plots of A) operation time, B) postoperative bile leakage, and C) intraoperative transfusion rates between left-side hepatectomy (LH) and right-side hepatectomy (RH). Fig. S5. Forest plots of A) overall postoperative morbidity, B) major postoperative morbidity, C) post-hepatectomy liver failure (PHLF), and postoperative bile leakage between left-side hepatectomy (LH) and right-side hepatectomy (RH). Fig. S6. Forest plots of A) overall postoperative mortality and B) in-hospital mortality (or perioperative motility) between left-side hepatectomy (LH) and right-side hepatectomy (RH). Fig. S7. Funnel plot of A) overall survival and B) R0 resection. [file 12957_2023_3037_MOESM2_ESM.zip › S4.tif]

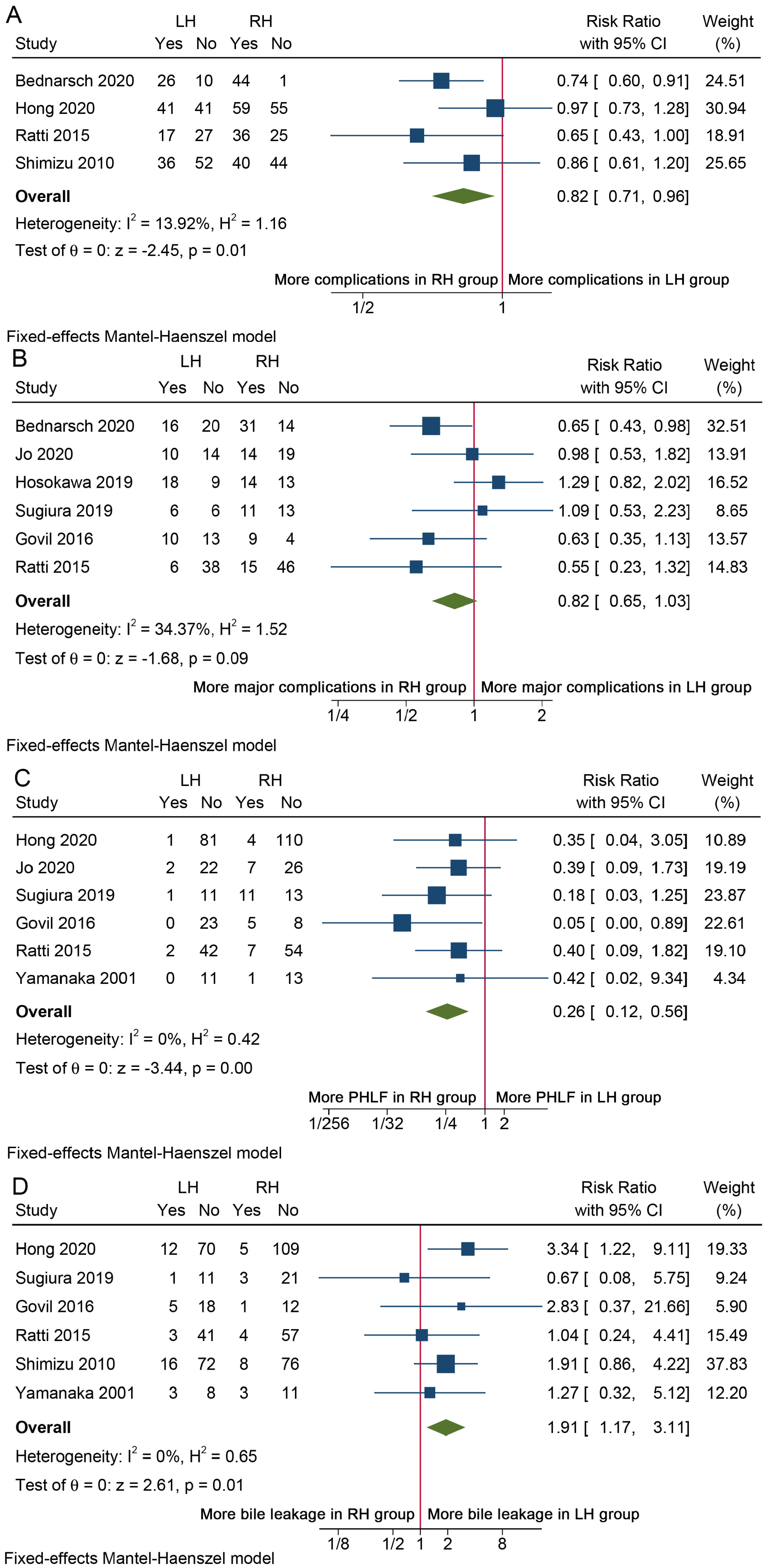

Supplement: Supplementary file 2 — Additional file 2: Fig. S1. Forest plot for the 1-year, 3-year and 5-year survival rates of patients with Hilar cholangiocarcinoma (HCCA) between left-side hepatectomy (LH) and right-side hepatectomy (RH). A 1-year survival rate. B 3-year survival rate. C 5-year survival rate. Fig. S2. Forest plot for the 1-year, 3-year and 5-year disease-free survival rate of patients with Hilar cholangiocarcinoma (HCCA) between left-side hepatectomy (LH) and right-side hepatectomy (RH). A 1-year disease-free survival rate. B 3-year disease-free survival rate. C 5-year disease-free survival rate. Fig. S3. Forest plots of A) preoperative total bilirubin levels, B) preoperative biliary drainage and C) portal vein embolization (PVE) between left-side hepatectomy (LH) and right-side hepatectomy (RH). Fig. S4. Forest plots of A) operation time, B) postoperative bile leakage, and C) intraoperative transfusion rates between left-side hepatectomy (LH) and right-side hepatectomy (RH). Fig. S5. Forest plots of A) overall postoperative morbidity, B) major postoperative morbidity, C) post-hepatectomy liver failure (PHLF), and postoperative bile leakage between left-side hepatectomy (LH) and right-side hepatectomy (RH). Fig. S6. Forest plots of A) overall postoperative mortality and B) in-hospital mortality (or perioperative motility) between left-side hepatectomy (LH) and right-side hepatectomy (RH). Fig. S7. Funnel plot of A) overall survival and B) R0 resection. [file 12957_2023_3037_MOESM2_ESM.zip › S5.tif]

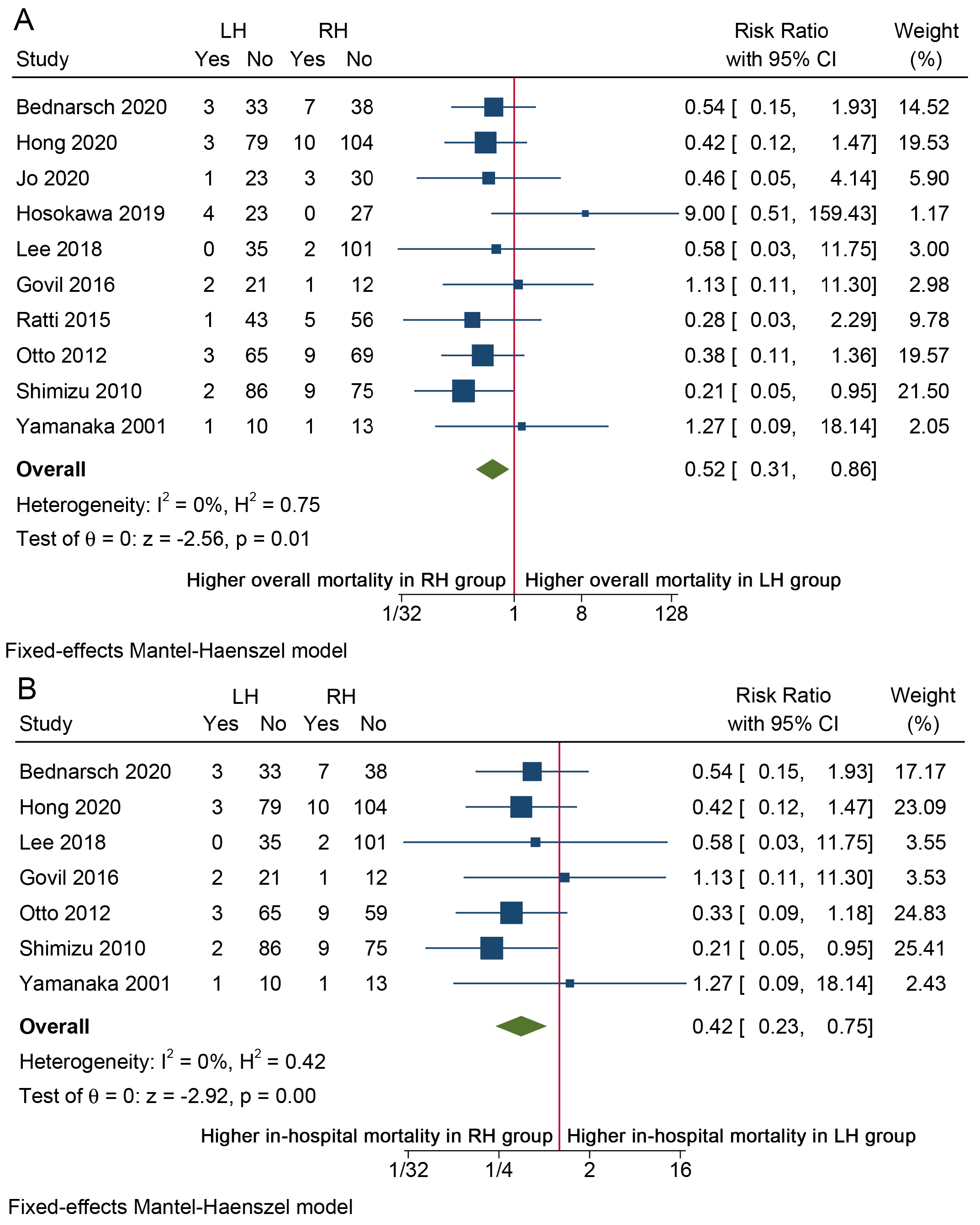

Supplement: Supplementary file 2 — Additional file 2: Fig. S1. Forest plot for the 1-year, 3-year and 5-year survival rates of patients with Hilar cholangiocarcinoma (HCCA) between left-side hepatectomy (LH) and right-side hepatectomy (RH). A 1-year survival rate. B 3-year survival rate. C 5-year survival rate. Fig. S2. Forest plot for the 1-year, 3-year and 5-year disease-free survival rate of patients with Hilar cholangiocarcinoma (HCCA) between left-side hepatectomy (LH) and right-side hepatectomy (RH). A 1-year disease-free survival rate. B 3-year disease-free survival rate. C 5-year disease-free survival rate. Fig. S3. Forest plots of A) preoperative total bilirubin levels, B) preoperative biliary drainage and C) portal vein embolization (PVE) between left-side hepatectomy (LH) and right-side hepatectomy (RH). Fig. S4. Forest plots of A) operation time, B) postoperative bile leakage, and C) intraoperative transfusion rates between left-side hepatectomy (LH) and right-side hepatectomy (RH). Fig. S5. Forest plots of A) overall postoperative morbidity, B) major postoperative morbidity, C) post-hepatectomy liver failure (PHLF), and postoperative bile leakage between left-side hepatectomy (LH) and right-side hepatectomy (RH). Fig. S6. Forest plots of A) overall postoperative mortality and B) in-hospital mortality (or perioperative motility) between left-side hepatectomy (LH) and right-side hepatectomy (RH). Fig. S7. Funnel plot of A) overall survival and B) R0 resection. [file 12957_2023_3037_MOESM2_ESM.zip › S6.tif]

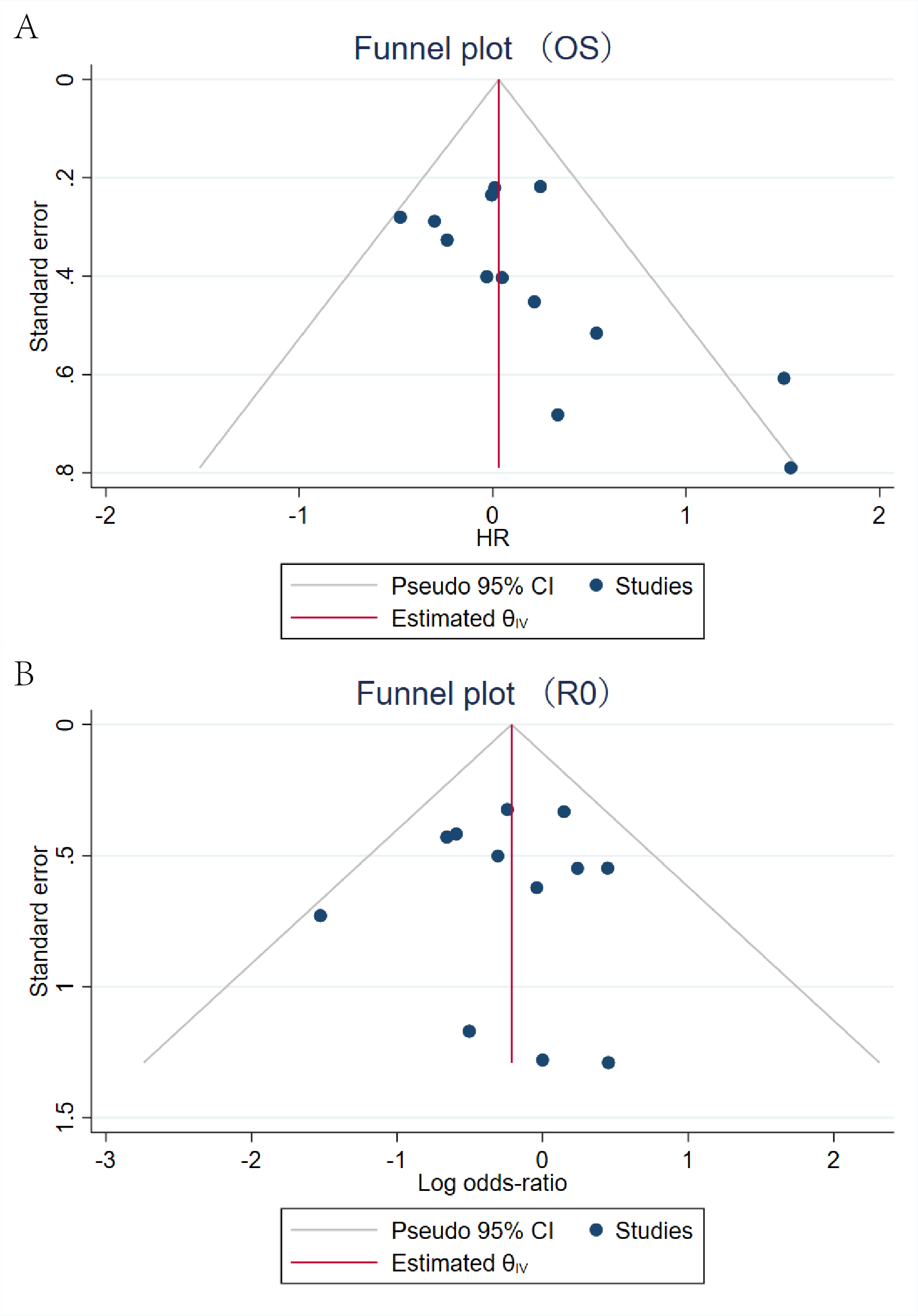

Supplement: Supplementary file 2 — Additional file 2: Fig. S1. Forest plot for the 1-year, 3-year and 5-year survival rates of patients with Hilar cholangiocarcinoma (HCCA) between left-side hepatectomy (LH) and right-side hepatectomy (RH). A 1-year survival rate. B 3-year survival rate. C 5-year survival rate. Fig. S2. Forest plot for the 1-year, 3-year and 5-year disease-free survival rate of patients with Hilar cholangiocarcinoma (HCCA) between left-side hepatectomy (LH) and right-side hepatectomy (RH). A 1-year disease-free survival rate. B 3-year disease-free survival rate. C 5-year disease-free survival rate. Fig. S3. Forest plots of A) preoperative total bilirubin levels, B) preoperative biliary drainage and C) portal vein embolization (PVE) between left-side hepatectomy (LH) and right-side hepatectomy (RH). Fig. S4. Forest plots of A) operation time, B) postoperative bile leakage, and C) intraoperative transfusion rates between left-side hepatectomy (LH) and right-side hepatectomy (RH). Fig. S5. Forest plots of A) overall postoperative morbidity, B) major postoperative morbidity, C) post-hepatectomy liver failure (PHLF), and postoperative bile leakage between left-side hepatectomy (LH) and right-side hepatectomy (RH). Fig. S6. Forest plots of A) overall postoperative mortality and B) in-hospital mortality (or perioperative motility) between left-side hepatectomy (LH) and right-side hepatectomy (RH). Fig. S7. Funnel plot of A) overall survival and B) R0 resection. [file 12957_2023_3037_MOESM2_ESM.zip › S7.tif]
